# The Effects of Corrosion and Fire Damage on the Steel-Bolted T-Section Connections Used in the Steel Construction

**DOI:** 10.1021/acsomega.3c04130

**Published:** 2023-10-30

**Authors:** Mahyar Maali, Mahmut Kiliç, Merve Sagiroglu Maali, Abdulkadir Cüneyt Aydın

**Affiliations:** † Faculty of Engineering and Architecture, Civil Engineering Department, Erzurum Technical University, 25050 Erzurum, Turkey; ‡ Maali Steel Company, Atateknokent, 25030 Erzurum, Turkey; § Engineering Faculty, Department of Civil Engineering, Ataturk University, 25030 Erzurum, Turkey; ∥ Academy Sağlık Hiz. Müh. İnş. Taah. Elekt. Yay. Company, Atateknokent, 25030 Erzurum, Turkey

## Abstract

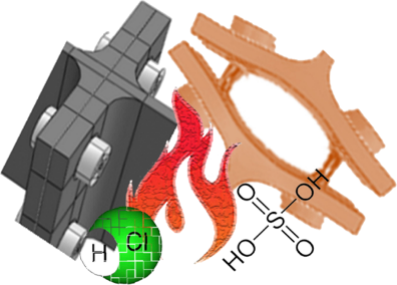

The most common type of connection in steel structures is the T-section connection. Steel structures can be damaged due to environmental effects such as earthquakes, fires, corrosion, etc., in real applications used in industry. Therefore, in this study, the corrosion and fire condition effects that occur in the T-section connection on the behavior of the connection zone were investigated. The study was carried out with 18 T-section connections with various corrosion (hydrochloric acid and sulfuric acid) in different layers at 5, 10, 15, and 20% corrosion levels, and fire (ISO834) conditions after corrosion have been evaluated and compared. T-section-bolted joints examined in the study were produced using IPE300 standard profiles. In the first part of the study, the behavior of the 18 T-section connections under an axial tensile load has been determined experimentally. The second part created a finite element model with the ABAQUS program for all models. It was seen that the finite element model analysis results converged with the results obtained as a result of the experimental study. As a result, compared to H_2_SO_4_ corrosion, HCl corrosion has little effect on the load–deformation characteristics of the connections. Also, if corrosion specimens are exposed to fire, then the connections will change from semirigid to hinged.

## 1 Introduction

**Figure 1:**
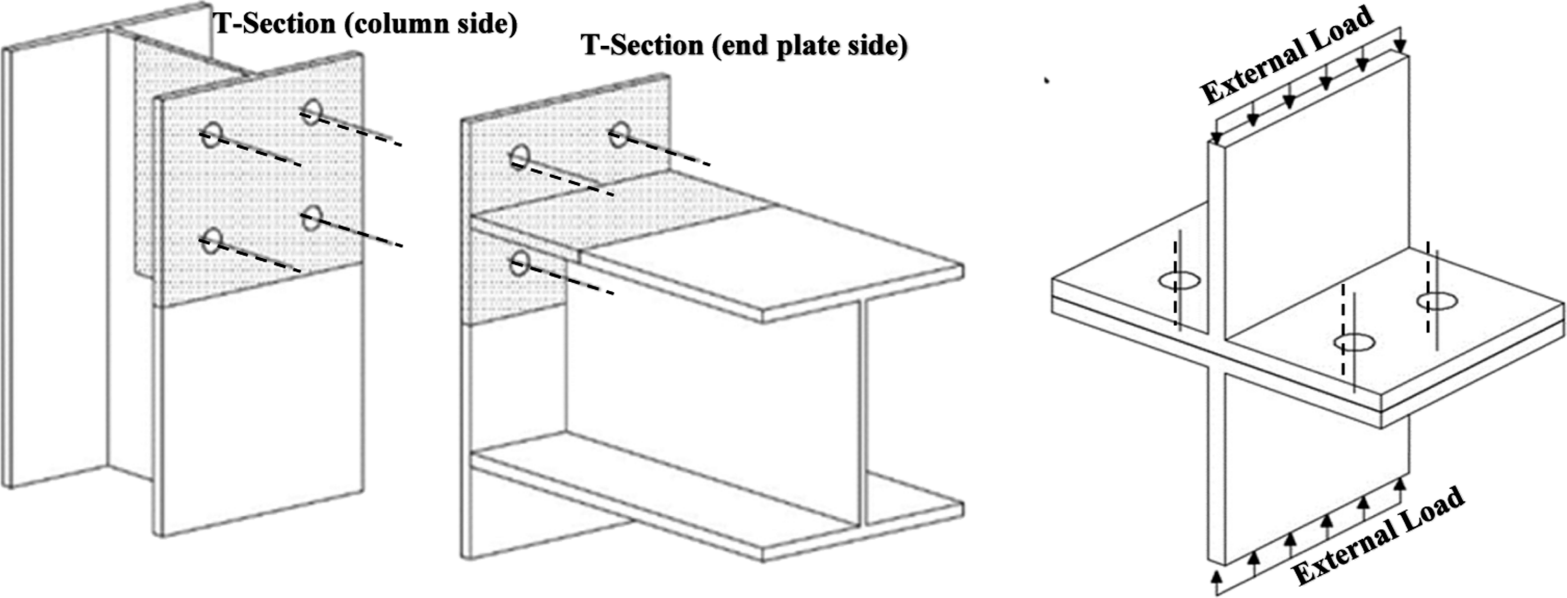
T-section model.

Steel structures are mainly preferred as construction materials in industrial buildings, business centers, and shopping centers. Besides the advantages of using steel material, there are also disadvantages: steel structures can be damaged due to environmental effects such as earthquakes, fires,^1−10^ corrosion, etc. Corrosion causes the surface to be separated into layers by the oxidation of steel structural elements due to electrochemical reactions and mass losses in the element.^11−13^ Also, the moment the fire reaches 600 degrees in a steel element, the steel loses its strength.^10^ Steel and similar metals tend to react with their environment’s elements and turn into compounds.^14^ The most common corrosion types are known as uniform corrosion (homogeneous, evenly distributed), galvanic corrosion, pitting corrosion, crevice corrosion, selective corrosion, intergranular corrosion, and erosion-corrosion.^15−18^ To eliminate the losses in load-carrying capacities, various studies have been carried out in the literature on the steel elements that have undergone corrosion and loss of section.^19−23^ Also, the most critical zone in steel buildings is the connection zone; research on various connections has been done in the literature, and research is still being done on the behavior of connections after corrosion 39-4and fire. As a result, one of these connections is the T-section beam-column connection;^24−27^ much research has been done in the literature, but there is very little research on the T-section beam-column connection after the fire and corrosion. Figure 1 shows that the T-section accounts for the column flange’s deformation and the end plate’s bending in the case of an extended end plate-bolted connection.^28^ Because the column flange is unstiffened, the T-section on the column side is orientated at right angles to the end plate T-section. The models for the column and the end plate sides are different. The T-section elements on the column flange side are generally hot-rolled profiles.

Some numerical and experimental studies from the literature are given below. Crosti^29^ presented the performance of steel structures under the effect of fire. Aziz et al.^30^ presented experimental and numerical studies on the fire performance of typical steel beams used in bridges. Petrina^31,32^ discussed the numerical simulations performed on the substructure of a steel building, namely, the column-beam end plate connection. Łukomski et al.^33^ presented the fire resistance test results for unprotected steel beams in EN 1993-1-2. Wong^34^ obtained the temperature distribution of a partially heated steel element using a simple finite difference scheme with parametrically coded generic elements. Li et al.^35^ conducted a study to obtain continuously effective thermal conductivity and fire tests of intumescent coatings with different properties based on comprehensive analysis data. So, the present study aims to investigate beam-to-column steel experimentally bolted T-section connections under different postfire conditions. T-section steel connection in the present study produced using the IPE standard profiles examined in the current study differs from those reported in the literature produced by welding plates. Thus, it is expected that problems such as breaking points that occur at the welds of the connections and the low strength could be overcome. On the other hand, more information about the behavior of these elements is needed to use weld-less T-connections. However, T-section connections with IPE standard profiles examined in this study are not mentioned or discussed in Eurocode 3.^36^ Additionally, this study examined the corrosion (hydrochloric acid and sulfuric acid) and fire effects, two critical disadvantages of steel structures. The load–deformation characteristic values and failure modes of the semirigid behavior T-section connections with various corrosion (hydrochloric acid and sulfuric acid) in different layers at 5, 10, 15, and 20% corrosion levels have been evaluated and compared. Also, after being subjected to corrosion, it was exposed to fire, one of the events that is not in the literature and is very likely; therefore, it was tested. Eighteen connections in the three groups were tested. In the experimental study, I steel sections (IPE300) were preferred because they are widely used in the fields of automotive, machinery, agricultural tools, space roof, ship construction, industrial buildings, warehouses, workshops, factories, wharves, coast, offshore, transportation, tourism buildings, etc. The test elements were made of S235JR steel, which is widely used in industry.

## 2 Experimental Investigation

### 2.1 Test Specimens

**Figure 2:**
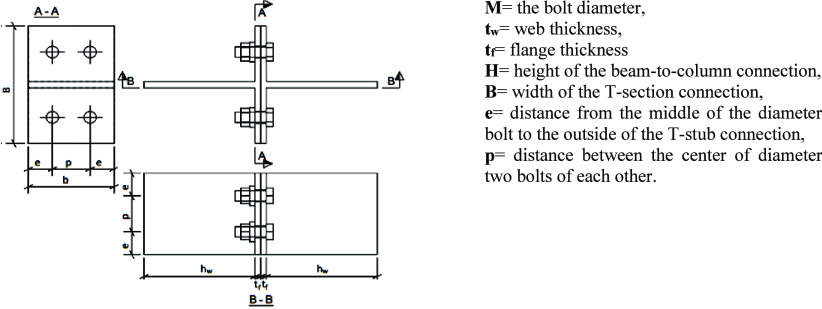
Geometries of T-section specimens and the description of nomenclatures.

This paper developed 18 experimental models to predict the behavior of bolted T-section connections under different corrosion and fire conditions under static loading in three groups (T300 sections cut from the IPE300 standard profile). The geometric properties of all specimens are listed in Figure 2 and Table 1. The behaviors of T-section connections were compared within their groups. Also, reference specimens for each cross section were examined for different corrosion and fire. Due to geometrical restrictions, T-sections were bonded with multiple bolts in a single row from their edges. The connections were subjected to tension parallel to the bolt axes. Thus, we aimed to obtain the tensile behavior of the connections and observe the formation of plastic hinges. The load–deformation characteristic values and failure modes of the semirigid behavior T-section connections with various corrosion (hydrochloric acid and sulfuric acid) in different layers at 5, 10, 15, and 20% corrosion levels have been evaluated and compared. Also, after being subjected to corrosion, it was exposed to fire, one of the events that is not in the literature and is very likely, so it was tested. Specimens connected through flanges were designed to fail according to plastic collapse mode-2, defined in EN 1993-1-8 part 6.^36^

**Table 1:** Test Specimen Properties^a^

**group**	**specimen**	**fire**	**hydrochloric acid (HCl)**	**sulfuric acid (H_2_SO_4_)**	**bolt**	***t*_w_(mm)**	***t***_**f**_**(mm)**	***H*(2*h*_w_** **+ 2*t*** _**f**_**)(mm)**	***B* (mm)**	*b* (2*e* + *P*) (mm)	***e* (mm)**	***p* (mm)**
HCl	HCl-5%		5%		M16	7.07	10.67	559.94	150	150	50	100
	HCl-10%		10%			6.94	10.54	559.68				
	HCl-15%		15%			6.75	10.35	559.3				
	HCl-20%		20%			6.40	10	558.6				
	F-HCl-5%	ISO 834	5%			6.92	10.52	559.64				
	F-HCl-10%	ISO 834	10%			6.91	10.51	559.62				
	F-HCl-15%	ISO 834	15%			6.70	10.3	559.2				
	F-HCl-20%	ISO 834	20%			6.35	9.95	558.5				
H_2_SO_4_	H_2_SO_4_-5%			5%		6.54	10.14	558.88				
	H_2_SO_4_-10%			10%		6.51	10.11	558.82				
	H_2_SO_4_-15%			15%		6.46	10.06	558.72				
	H_2_SO_4_-20%			20%		6.10	9.7	558				
	F-H_2_SO_4_-5%	ISO 834		5%		6.31	9.91	558.42				
	F-H_2_SO_4_-10%	ISO 834		10%		6.48	10.08	558.76				
	F-H_2_SO_4_-15%	ISO 834		15%		6.21	9.81	558.22				
	F-H_2_SO_4_-20%	ISO 834		20%		6	9.6	557.8				
perfect	P					7.1	10.7	560				
	F-P	ISO 834				7	10.6	559.8				

a HCl: hydrochloric acid; H_2_SO_4_: sulfuric acid; F: fire application; P: perfect model without corrosion and fire.

### 2.2 Mechanical Properties

**Figure 3:**
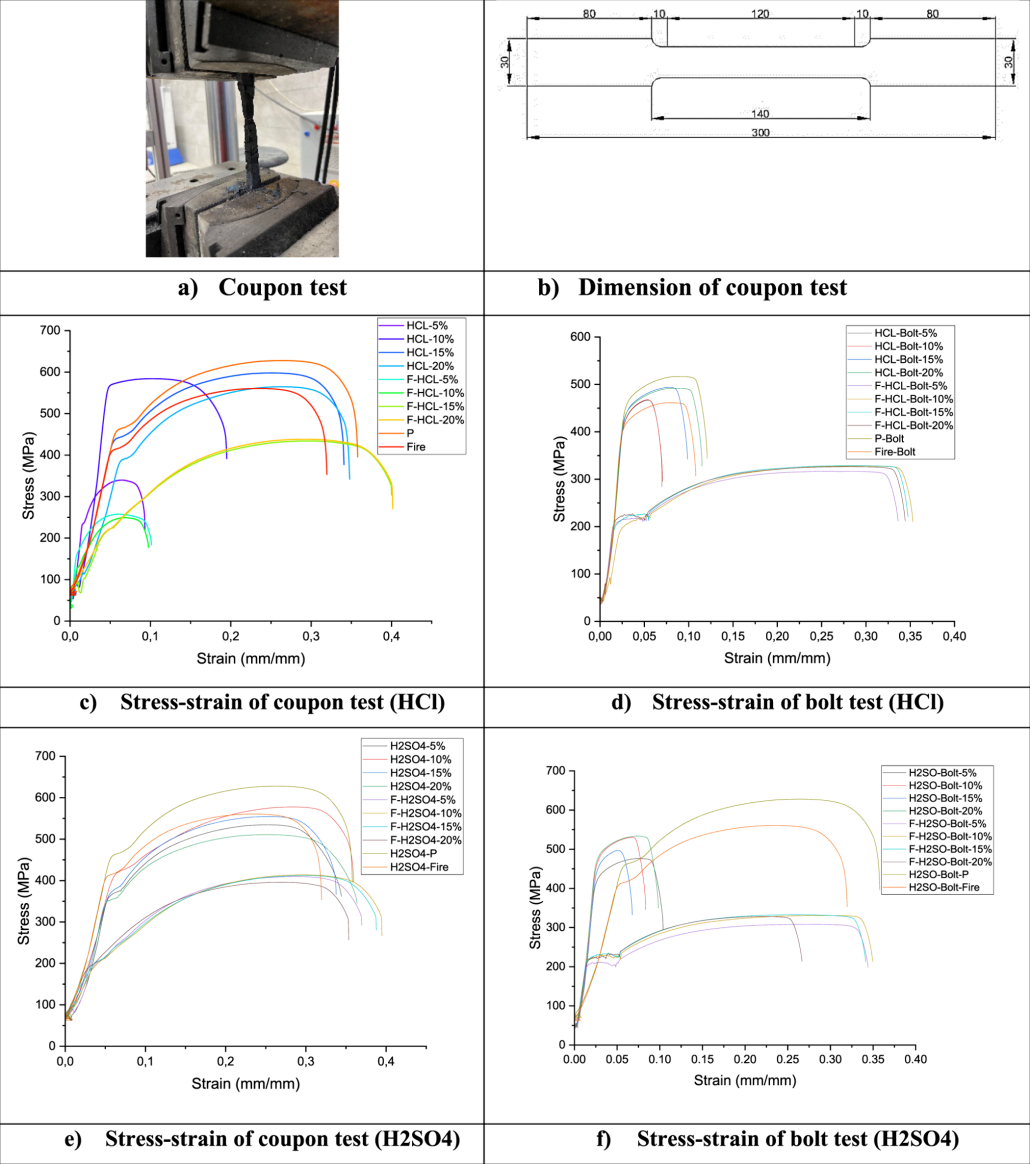
Test setup of tensile coupon test and stress–strain curve.

A total of 54 tensile coupon tests were carried out to obtain the mechanical properties of the test specimens. The coupon tension test of the structural steel material was performed, complying with UNE-EN 10002-1.^37^ These were then tested using a Bestmark machine with a 300 kN capacity (Ataturk University, Turkey). The test coupons are shown in Figure 3. The active matrix of the axial tensile tests performed to determine the mechanical properties of the materials used in the experimental study is presented in Table 2. The average characteristic values for structural steels and bolts (8.8) are listed in Table 2. Next, each bolt (8.8) was tested under tension to determine the bolt material’s mechanical properties, per ISO 898-1999.^38^

**Table 2:** Average Characteristic Values for Structural Steels and Bolt (M16-8.8)^a^

	**HCl**				**fire-HCl**				**steel**		**H_2_SO_4_**				**fire-H_2_SO_4_**			
**steel**	5%	10%	15%	20%	5%	10%	15%	20%	P	fire	5%	10%	15%	20%	5%	10%	15%	20%
*E* (MPa)	203214	204444	201456	200125	203216	204211	203211	201111	206301	204215	201415	203412	201425	200969	204445	204549	206525	206211
*f_y_* (MPa)	239.84	573.69	443.53	392.01	165.32	132.15	210.46	217.62	399.90	300	370.18	423.12	380.63	356.29	196.47	193.54	199.52	187.63
*f*_*u*_ (MPa)	339.81	584.20	598.140	564.72	257.85	249.40	434.07	438.13	599.85	485.80	534.68	577.79	554.59	510.85	409.62	413.919	412.17	395.93
ρ*_y_* = *f*_*y*_/*f*_*u*_	0.71	0.98	0.74	0.69	0.64	0.53	0.48	0.50	0.67	0.62	0.69	0.73	0.69	0.70	0.48	0.47	0.48	0.47

	**HCl**				**fire-HCl**				**bolt**		**H_2_SO_4_**				**fire-H_2_SO_4_**			
**bolt**	5%	10%	15%	20%	5%	10%	15%	20%	P	fire	5%	10%	15%	20%	5%	10%	15%	20%
*f_y_* (MPa)	438.34	442.52	451.18	448.28	205.85	189.68	207.46	214.42	540	492	433	483.82	473.26	496.34	206.72	219.63	219.52	218.41
*f*_*u*_ (MPa)	466.86	467.73	493.92	492.15	316.73	327.83	328.63	326.59	725.52	689.25	476.66	530.58	496.96	533.70	308.43	330.86	332.86	328.12
ρ*_y_* = *f*_y_/*f*_*u*_	0.94	0.95	0.91	0.91	0.65	0.58	0.63	0.66	0.74	0.71	0.91	0.91	0.95	0.93	0.67	0.66	0.66	0.66

a *E* = Young’s modulus; *f_y_* = static yield; *f_u_* = tensile stress.

### 2.3 Fire Application

**Figure 4:**
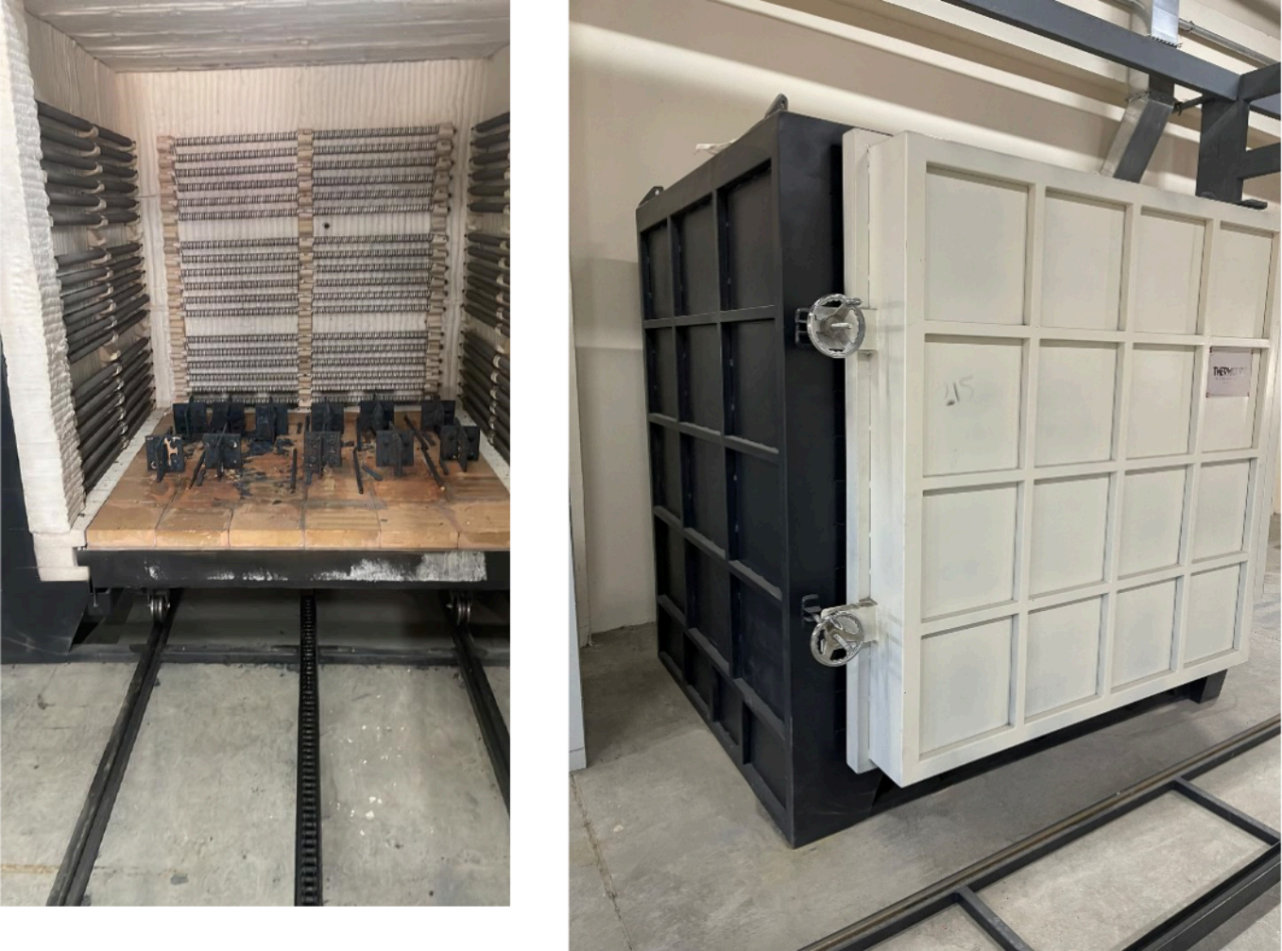
The furnace is used for fire exposure.

**Figure 5:**
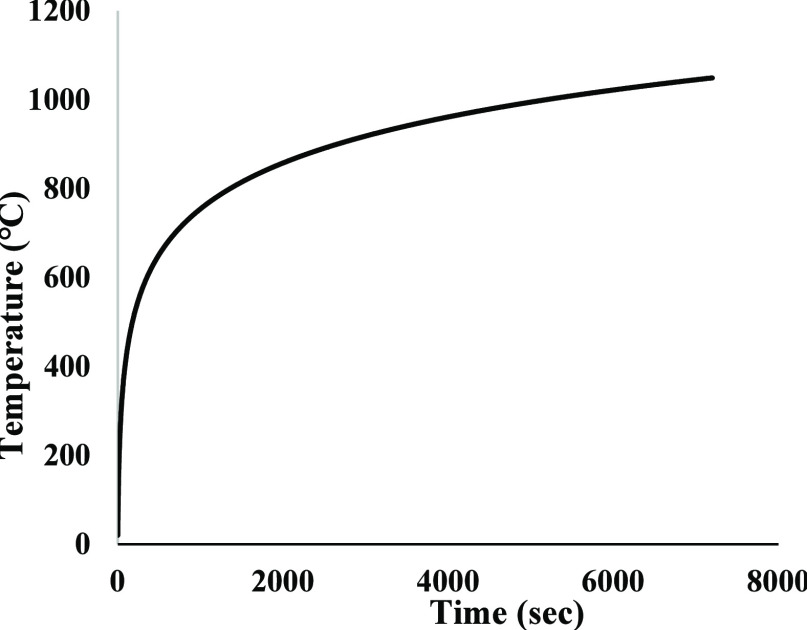
Standard fire curve (ISO834-fire).

The specimens were then exposed to predetermined high temperatures using a furnace (Figure 4). The fire curve (ISO834, Eurocode 1: Actions on structures Part 1–2: General actions—Actions on structures exposed to fire)^39^ was calculated using the following standard load-time graph equation:
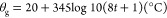
1where “θ_g_” is the gas temperature in the fire compartment (°C) and “*t*” is the time (min). The standard fire curve and the fire temperature values used are listed in Figure 5.

### 2.4 Testing and Loading Procedure

**Figure 6:**
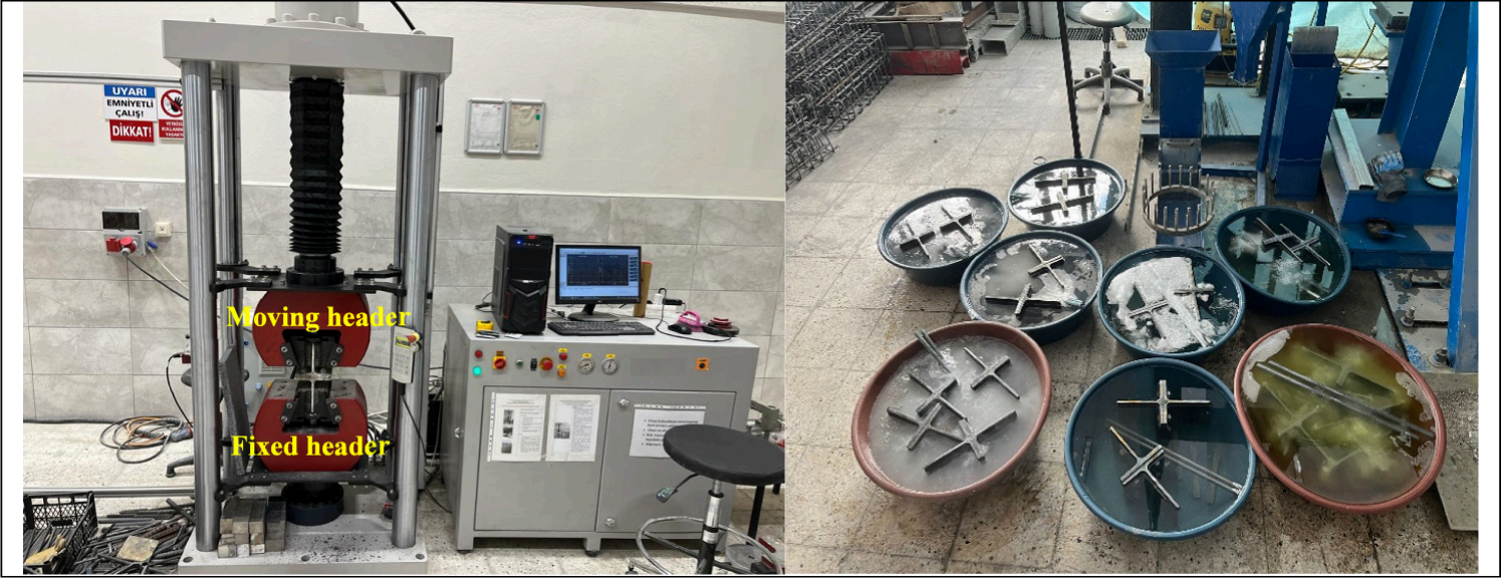
Test instruments and specimens exposed to corrosion.

These were then tested using a Bestmark machine with a 300 kN capacity and a torque span of 900 mm (Figure 6). Experiments were carried out under a loading rate of 6 MPa/s until the collapse of T-section connections. The specimens underwent a series of treatments before being placed in the test setup. First, the specimens were bolted. After the bolted specimens were exposed to the predetermined corrosion (Figure 6). Second, specimens were exposed to the predetermined fire in a high-temperature furnace (ISO834). Finally, the specimens were taken out of the furnace and left to cool down. In the current study, the characteristics and dimensions of the T-section specimens were determined according to Eurocode 3. Also, the load–deformation curve characteristics given in Eurocode 3 were used in all tests. The load–deformation curves obtained resistance, stiffness, deformation capacity, and energy dissipations.

## 3 Test Results and Discussion

**Figure 7:**
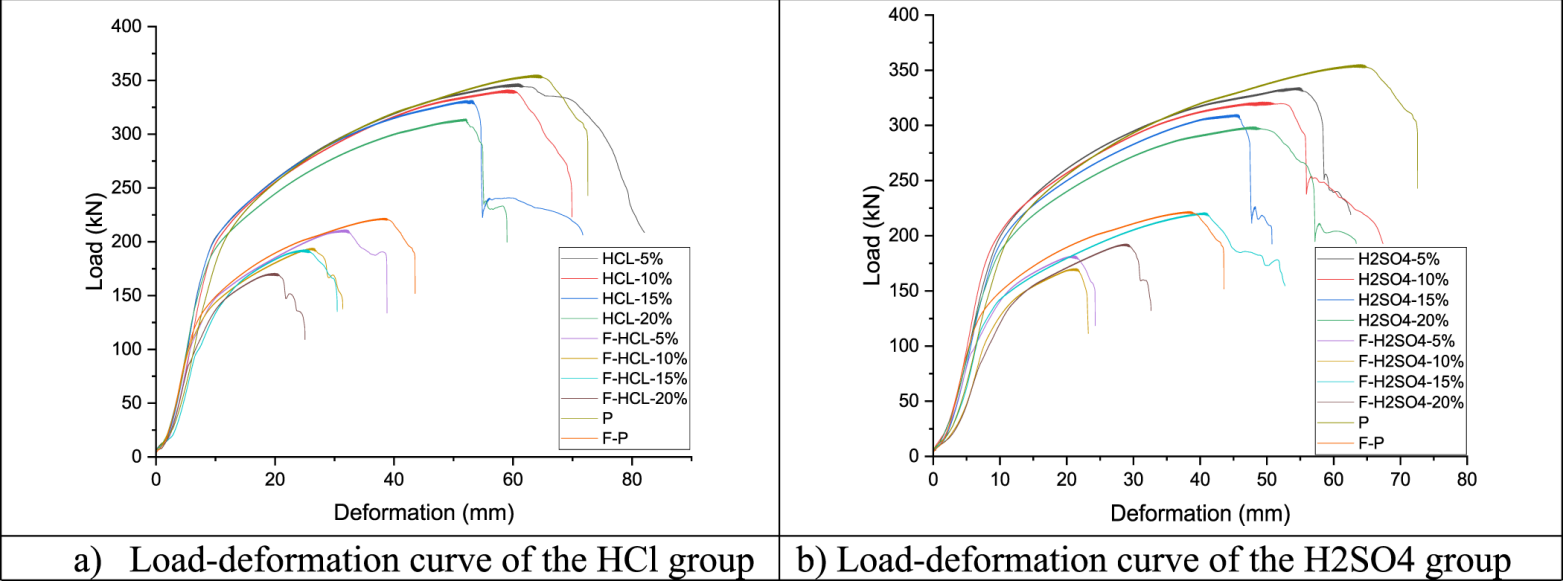
Load–deformation of the all-specimen test.

**Figure 8:**
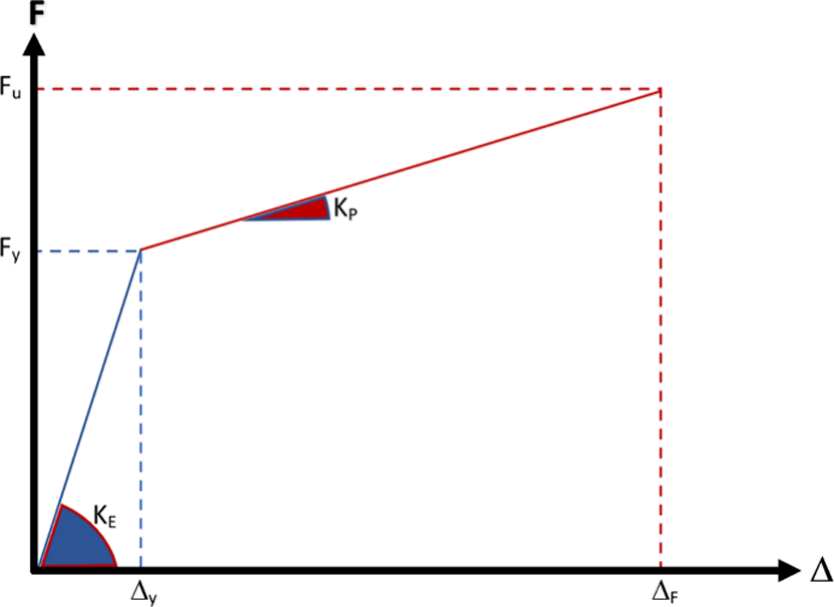
Determining of initial stiffness and post-limit stiffness.

The test results obtained for 18 specimens of steel-bolted T-section connections with different fire and corrosion conditions are given in Figure 7 and Table 3. Table 3 also presents the values obtained from the analysis of the test results. The force–strain curves obtained as a result of the experiments are also presented in Figure 8. Initial stiffness and post-limit stiffness values in the load–displacement curves obtained as a result of the experimental study were calculated, as shown in Figure 8. In addition, yield stress, maximum stress value, yield strain, and shear strain were determined from the load–strain curve.^39−41^

**Table 3:** Load–Deformation Characteristics for All Specimens

			**stiffness (kN/mm)**				
**group**	**specimen**	**maximum load (Fu) (kN)**	initial stiffness **(Ke)**	post-limit stiffness **(Kp)**	**Kp/Ke**	**deformation capacity Δ*f* (mm)**	**energy dissipation (kN mm)**
HCl	HCl-5%	347.10	3.4	0.28	12.14	82.09	14246.7
	HCl-10%	341.25	2.33	0.23	10.13	55.92	9541.35
	HCl-15%	331.53	2.82	0.26	10.85	57.38	9511.59
	HCl-20%	314.28	2.04	0.22	9.27	47.19	7415.4
	F-HCl-5%	211.21	1.77	0.28	6.32	38.83	4100.64
	F-HCl-10%	194.11	1.23	0.22	5.59	25.11	2437.05
	F-HCl-15%	192.67	1.05	0.22	4.77	24.36	2346.72
	F-HCl-20%	170.80	1.33	0.29	4.59	25.04	2138.42
H_2_SO_4_	H_2_SO_4_-5%	334.36	2.59	0.22	11.77	50.047	8366.86
	H_2_SO_4_-10%	321.32	3.70	0.34	10.88	53.92	8662.79
	H_2_SO_4_-15%	309.96	2.16	0.30	7.2	40.61	6293.73
	H_2_SO_4_-20%	298.86	2.05	0.29	7.07	50.70	7576.10
	F-H_2_SO_4_-5%	220.92	1.58	0.30	5.27	42.17	4658.09
	F-H_2_SO_4_-10%	192.58	1.42	0.32	4.43	26.12	2515.09
	F-H_2_SO_4_-15%	181.92	1.093	0.25	4.38	19.47	1770.99
	F-H_2_SO_4_-20%	170.26	1.08	0.26	4.15	18.59	1582.85
perfect	P	355.20	3.93	0.28	14.03	58.068	10312.88
	F-P	242.55	3.73	0.29	12.86	38.96	4724.87

### 3.1 HCl Group

The maximum load value (Fu) is given in Table 3; the HCl corrosion value is increased from 5 to 20%, and the maximum load value is decreased by about 10.44%. That is, the maximum load value decreases as the corrosion value increases. Also, specimens with HCl corrosion were compared with the P specimen in group 3. The maximum load values decreased for HCl-5%, HCl-10%, HCl-15%, and HCl-20% specimens, about 2.3, 4.08, 7.14, and 13.20 for the perfect model, respectively.

The HCl corrosion with fire condition value increased from 5 to 20%; the maximum load value decreased by 23.65%. The maximum load value decreases as the corrosion with the fire value increases. Also, specimens with HCl corrosion were compared with the F-P specimen in group 3. The maximum load values decreased for F-HCl-5%, F-HCl-10%, F-HCl-15%, and F-HCl-20% specimens, about 14.83, 24.95, 25.88, and 42.01% for the F-P model, respectively. If the F-P specimen is compared with those only exposed to corrosion, the corrosion rate should increase by about 30% to equal the maximum load value of the F-P specimen.

**Figure 9:**
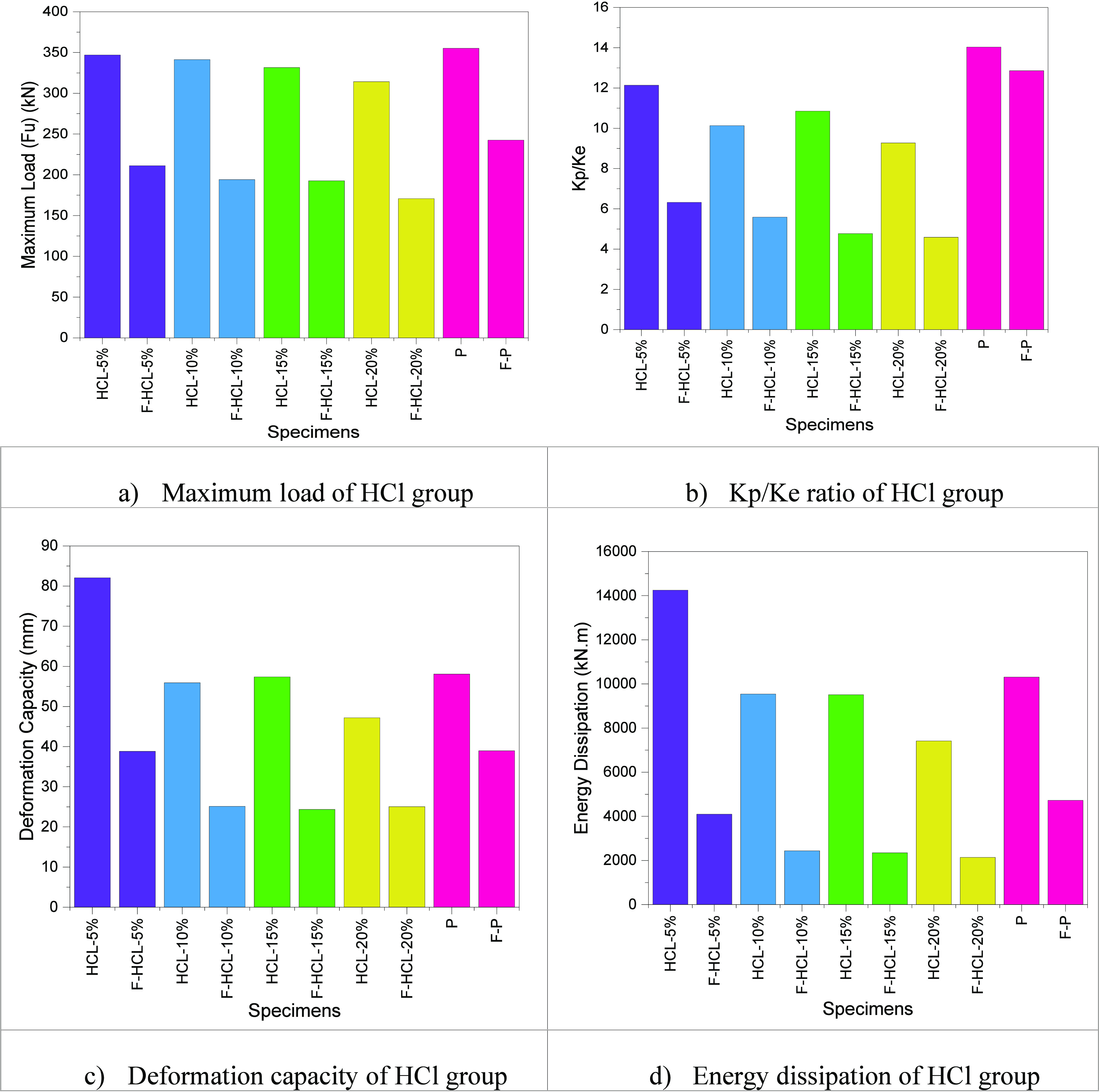
Comparing the HCl group.

Figure 9 shows the corrosion with fire condition specimens compared to the corrosion specimen. The maximum load values of the HCl-5%, HCl-10%, HCl-15%, and HCl-20% specimens compared with F-HCl-5%, F-HCl-10%, F-HCl-15%, and F-HCl-20% specimens decreased by 64.34, 75.80, 72.07, and 84%, respectively.

The stiffness ratio (Kp/Ke) value is given in Table 3; the HCl corrosion value increased from 5 to 20%, and the stiffness ratio (Kp/Ke) value decreased by about 30.96%. The stiffness decreases as the HCl corrosion ratio increases from 5 to 20%. Also, specimens with HCl corrosion were compared with the P specimen in group 3. The stiffness ratio (Kp/Ke) values decreased for HCl-5%, HCl-10%, HCl-15%, and HCl-20% specimens, about 15.15, 38.49, 29.30, and 51.34% for the perfect model, respectively. In other words, as the HCl ratio increases, the semirigid behavior will decrease, exhibiting a hinged behavior.

The HCl corrosion with fire condition value increased from 5 to 20%, and the stiffness ratio (Kp/Ke) value decreased by about 37.69%. The maximum load value decreases as the corrosion with fire value increases. Also, specimens with HCl corrosion were compared with the F-P specimen in group 3. The stiffness ratio (Kp/Ke) value decreased for F-HCl-5%, F-HCl-10%, F-HCl-15%, and F-HCl-20% specimens, about 103.48, 130.05, 169.60, and 180.17% for the F-P model, respectively.

Figure 9 shows the corrosion with fire condition specimens compared to the corrosion specimen. The stiffness ratio (Kp/Ke) values of the HCl-5%, HCl-10%, HCl-15%, and HCl-20% specimens compared with F-HCl-5%, F-HCl-10%, F-HCl-15%, and F-HCl-20% specimens decreased by 92.08, 81.22, 127.46, and 101.96%, respectively. In general, if the fire acts together with the corrosion, then the behavior of the connections can change from semirigid to hinged.

The deformation capacity (Δ*y*) value is given in Table 3; the HCl corrosion value is increased from 5 to 20%, and the deformation capacity (Δ*y*) value decreases by about 73.95%. As the HCl corrosion ratio increases from 5 to 20%, the deformation capacity (Δ*y*) decreases. Also, specimens with HCl corrosion were compared with the P specimen in group 3. The deformation capacity (Δ*y*) values decreased for HCl-10%, HCl-15%, and HCl-20% specimens, about 3.8, 1.19, and 23.05% for the perfect model, respectively. However, the deformation capacity (Δ*y*) value increased for the HCl-5% specimen, about 29.26% for the perfect model.

The HCl corrosion with fire condition value increased from 5 to 20%, and the deformation capacity (Δ*y*) value decreased by about 55.07%. The deformation capacity (Δ*y*) value decreases as the corrosion with fire value increases. Also, specimens with HCl corrosion were compared with the F-P specimen in group 3. The deformation capacity (Δ*y*) values decreased for F-HCl-5%, F-HCl-10%, F-HCl-15%, and F-HCl-20% specimens, about 0.33, 55.16, 59.93, and 55.59% for the F-P model, respectively.

Figure 9 shows the corrosion with fire condition specimens compared to the corrosion specimen. The deformation capacity (Δ*y*) values of the HCl-5%, HCl-10%, HCl-15%, and HCl-20% specimens compared with F-HCl-5%, F-HCl-10%, F-HCl-15%, and F-HCl-20% specimens decreased by 111.41, 122.70, 135.55, and 88.45%, respectively. In general, if the fire acts together with the corrosion, then the behavior of the connections can change from semirigid to hinged.

The energy dissipation value is given in Table 3; the HCl corrosion value is increased from 5 to 20%, and the energy dissipation value decreased by about 92.12%. The energy dissipation value decreases as the HCl corrosion ratio increases from 5 to 20%. Also, specimens with HCl corrosion were compared with the P specimen in group 3. The energy dissipation values decreased for HCl-10%, HCl-15%, and HCl-20% specimens, about 8.08, 8.42, and 39.07% for the perfect model, respectively. However, the energy dissipation value increased for the HCl-5% specimen, about 27.61% for the perfect model, respectively.

The HCl corrosion with fire condition value increased from 5 to 20%; the energy dissipation value decreased by about 91.76%. The energy dissipation value decreases as the corrosion with fire value increases. Also, specimens with HCl corrosion compared with the F-P specimen in group 3 decreased energy dissipation for F-HCl-5%, F-HCl-10%, F-HCl-15%, and F-HCl-20% specimens, about 15.22, 93.87, 101.34, and 120.95% for the F-P model, respectively.

Figure 9 shows the corrosion with fire condition specimens compared to the corrosion specimen. The energy dissipation values of the HCl-5%, HCl-10%, HCl-15%, and HCl-20% specimens compared with F-HCl-5%, F-HCl-10%, F-HCl-15%, and F-HCl-20% specimens decreased by 247.43, 291.51, 305.31, and 246.77%, respectively.

### 3.2 H_2_SO_4_ Group

The maximum load value (Fu) is given in Table 3; the H_2_SO_4_ corrosion value is increased from 5 to 20%, and the maximum load value is decreased by about 11.88%. That is, the maximum load value decreases as the corrosion value increases. Also, specimens with H_2_SO_4_ corrosion were compared with the P specimen in group 3. The maximum load values decreased for H_2_SO_4_-5%, H_2_SO_4_-10%, H_2_SO_4_-15%, and H_2_SO_4_-20% specimens, about 3.24, 10.54, 14.59, and 18.85% for the perfect model, respectively.

The H_2_SO_4_ corrosion with the fire condition value increased from 5 to 20%; the maximum load value decreased by about 29.75%. The maximum load value decreases as the corrosion with fire value increases. Also, specimens with H_2_SO_4_ corrosion were compared with the F-P specimen in group 3. The maximum load values decreased for F-H_2_SO_4_-5%, F-H_2_SO_4_-10%, F-H_2_SO_4_-15%, and F-H_2_SO_4_-20% specimens, about 9.79, 25.95, 33.33, and 42.46% for the F-P model, respectively.

**Figure 10:**
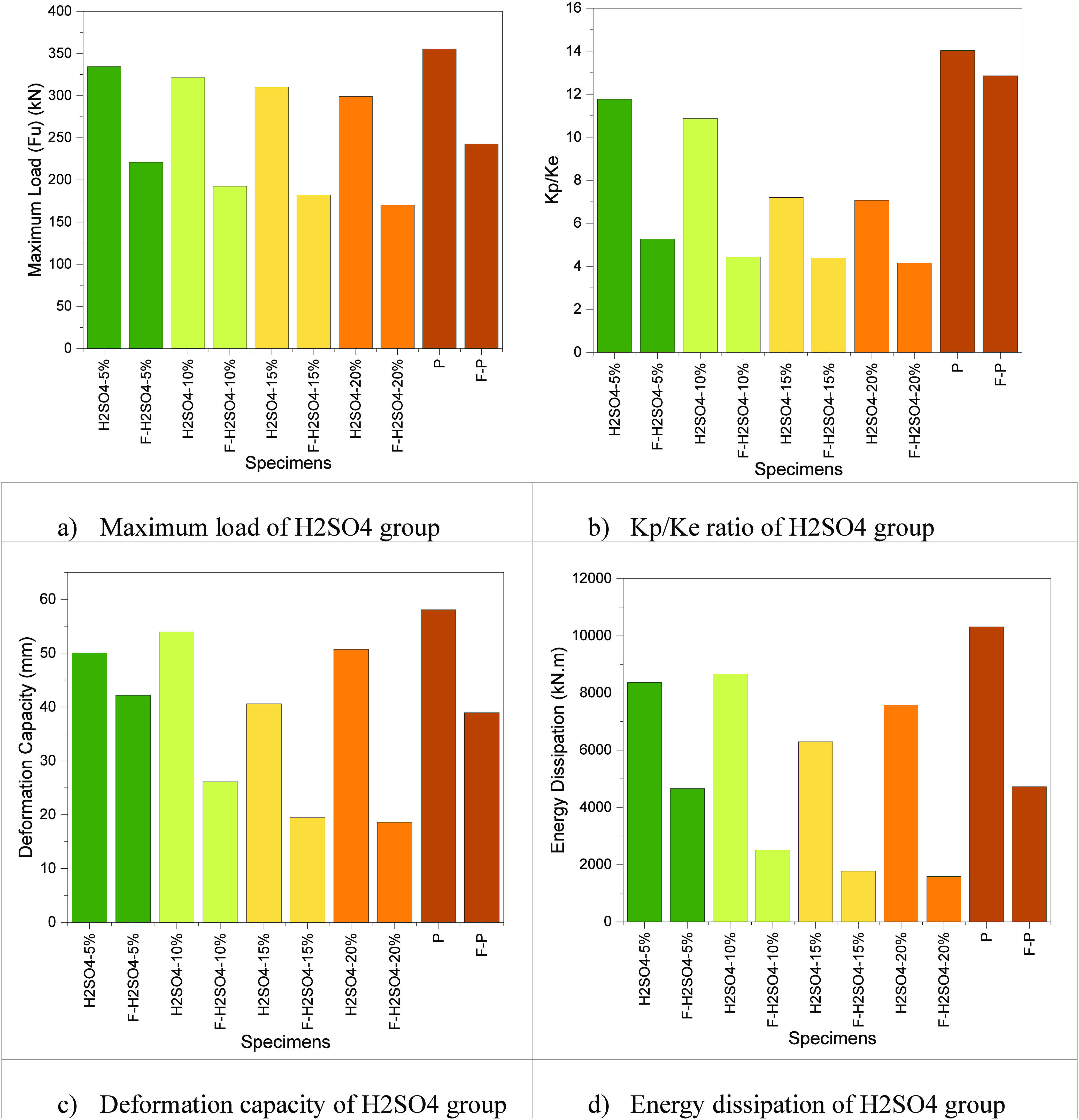
Comparing the H_2_SO_4_ group.

Figure 10 shows the corrosion with fire condition specimens compared to the corrosion specimen. The maximum load values of the H_2_SO_4_-5%, H_2_SO_4_-10%, H_2_SO_4_-15%, and H_2_SO_4_-20% specimens compared with F-H_2_SO_4_-5%, F-H_2_SO_4_-10%, F-H_2_SO_4_-15%, and F-H_2_SO_4_-20% specimens decreased by 51.34, 66.85, 70.38, and 75.53%, respectively.

The stiffness ratio (Kp/Ke) value is given in Table 3; the H_2_SO_4_ corrosion value is increased from 5 to 20%, and the stiffness ratio (Kp/Ke) value decreases by about 66.48%. The stiffness decreases as the H_2_SO_4_ corrosion ratio increases from 5 to 20%. Also, specimens with H_2_SO_4_ corrosion were compared with the P specimen in group 3. The stiffness ratio (Kp/Ke) values decreased for H_2_SO_4_-5%, H_2_SO_4_-10%, H_2_SO_4_-15%, and H_2_SO_4_-20% specimens, about 19.20, 28.95, 94.86, and 98.44% for the perfect model, respectively. In other words, as the H_2_SO_4_ ratio increases, the semirigid behavior will decrease, exhibiting a hinged behavior.

The H_2_SO_4_ corrosion with fire condition value increased from 5 to 20%, and the stiffness ratio (Kp/Ke) value decreased by about 26.98%. The maximum load value decreases as the corrosion with fire value increases. Also, specimens with H_2_SO_4_ corrosion were compared with the F-P specimen in group 3. The stiffness ratio (Kp/Ke) values decreased for F-H_2_SO_4_-5%, F-H_2_SO_4_-10%, F-H_2_SO_4_-15%, and F-H_2_SO_4_-20% specimens, about 144.02, 190.29, 193.61, and 209.87% for the F-P model, respectively.

Figure 10 shows the corrosion with fire condition specimens compared to the corrosion specimen. The stiffness ratio (Kp/Ke) values of the H_2_SO_4_-5%, H_2_SO_4_-10%, H_2_SO_4_-15%, and H_2_SO_4_-20% specimens compared with F-H_2_SO_4_-5%, F-H_2_SO_4_-10%, F-H_2_SO_4_-15%, and F-H_2_SO_4_-20% specimens decreased by 123.34, 145.59, 64.38, and 70.36%, respectively. In general, if the fire acts together with the corrosion, then the behavior of the connections can change from semirigid to hinged.

Specimens with H_2_SO_4_ corrosion were compared with the P specimen in group 3. The deformation capacity (Δ*y*) values increased for H_2_SO_4_-5%, H_2_SO_4_-10%, H_2_SO_4_-15%, and H_2_SO_4_-20% specimens, about 22.15, 27.74, 4.06, and 23.15% for the perfect model, respectively.

The H_2_SO_4_ corrosion with fire condition value is increased from 5 to 20%; the deformation capacity (Δ*y*) value decreased by about 126.84%. The deformation capacity (Δ*y*) value decreases as the corrosion with fire value increases. Also, specimens with H_2_SO_4_ corrosion were compared with the F-P specimen in group 3. The deformation capacity (Δ*y*) values decreased for F-H_2_SO_4_-10%, F-H_2_SO_4_-15%, and F-H_2_SO_4_-20% specimens, about 49.16, 100.10, and 109.57% for the F-P model, respectively

Figure 10 shows the corrosion with fire condition specimens compared to the corrosion specimen. The deformation capacity (Δ*y*) values of the H_2_SO_4_-5%, H_2_SO_4_-10%, H_2_SO_4_-15%, and H_2_SO_4_-20% specimens compared with F-H_2_SO_4_-5%, F-H_2_SO_4_-10%, F-H_2_SO_4_-15%, and F-H_2_SO_4_-20% specimens decreased by 18.68, 106.43, 108.58, and 172.72%, respectively. In general, if the fire acts together with the corrosion, then the behavior of the connections can change from semirigid to hinged.

The energy dissipation value is given in Table 3; the H_2_SO_4_ corrosion value is increased from 5 to 20%, and the energy dissipation value decreased by about 10.44%. As the H_2_SO_4_ corrosion ratio increases from 5 to 20%, the energy dissipation value decreases. Also, specimens with H_2_SO_4_ corrosion were compared with the P specimen in group 3. The energy dissipation values increased for H_2_SO_4_-5%, H_2_SO_4_-10%, H_2_SO_4_-15%, and H_2_SO_4_-20% specimens, about 43.53, 45.45, 24.92, and 37.63% for the perfect model, respectively.

The H_2_SO_4_ corrosion with fire condition value increased from 5 to 20%; the energy dissipation value decreased by about 194.28%. The energy dissipation value decreases as the corrosion with fire value increases. Also, specimens with H_2_SO_4_ corrosion compared with the F-P specimen in group 3 decreased energy dissipation for F-H_2_SO_4_-5%, F-H_2_SO_4_-10%, F-H_2_SO_4_-15%, and F-H_2_SO_4_-20% specimens, about 1.43, 87.86, 166.79, and 198.50% for the F-P model, respectively.

Figure 10 shows the corrosion with fire condition specimens compared to the corrosion specimen. The energy dissipation values of the H_2_SO_4_-5%, H_2_SO_4_-10%, H_2_SO_4_-15%, and H_2_SO_4_-20% specimens compared with F-H_2_SO_4_-5%, F-H_2_SO_4_-10%, F-H_2_SO_4_-15%, and F-H_2_SO_4_-20% specimens decreased by 79.62, 244.43, 255.38, and 378.63%, respectively.

### 3.3 Comparison of the HCl, H_2_SO_4_, and Perfect Groups

**Figure 11:**
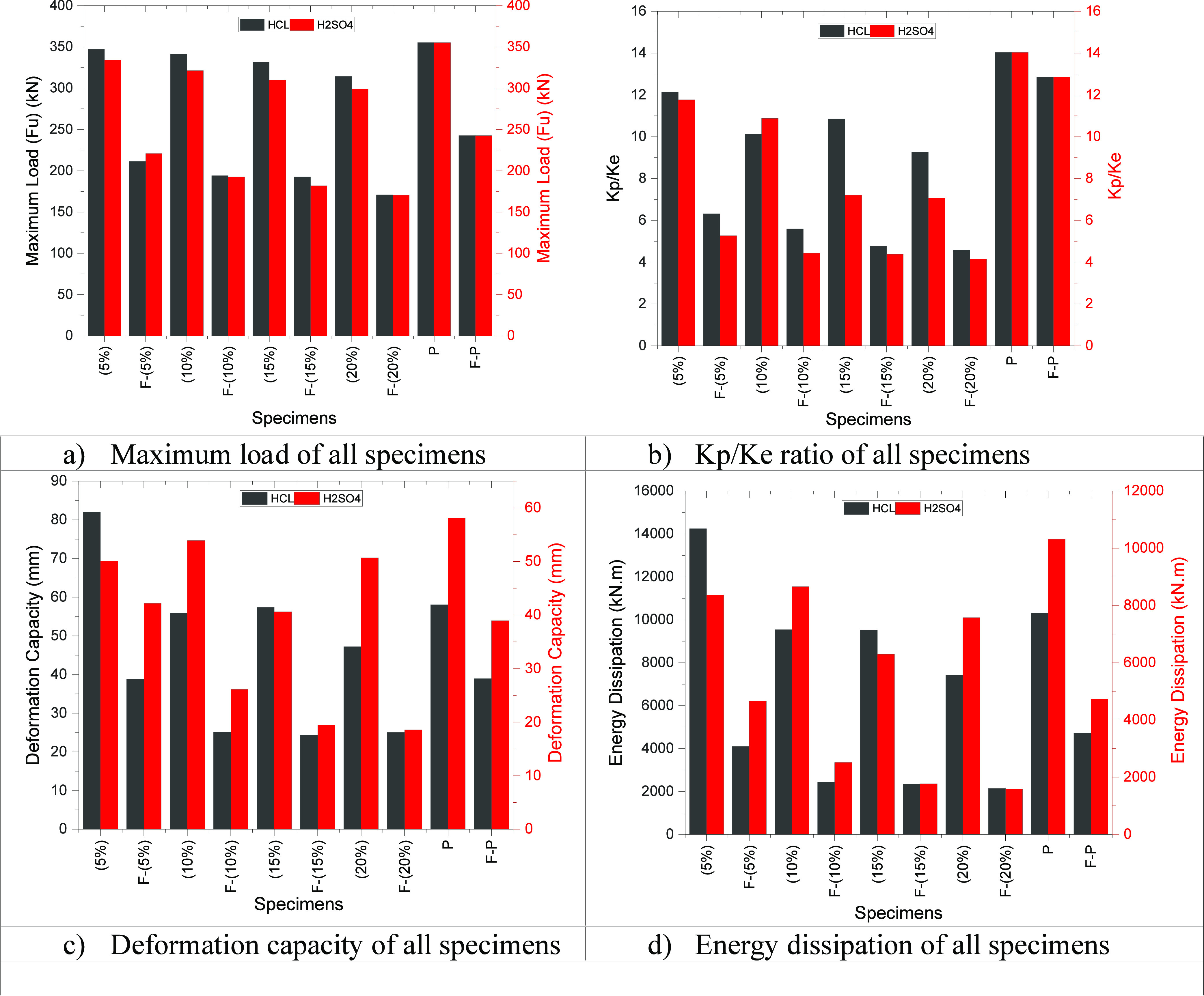
Comparison of the HCl, H_2_SO_4_, and perfect groups.

Table 4 and Figure 11 show a comparison of the HCl, H_2_SO_4_, and perfect groups with each other. For example, Table 3 shows that the maximum load value decreased by about 0.32–6.51%, according to the H_2_SO_4_ specimens of the HCl specimens. In other words, in H_2_SO_4_ corrosion, the maximum load value decreases considerably compared with HCl corrosion. So, if the joint is exposed to H_2_SO_4_ corrosion, then its semirigidity behavior approaches that of hinged behavior. Also, the maximum load value decreased by about 31%, according to the F-P specimen of the P specimen.

**Table 4:** Comparison of the HCl, H_2_SO_4_, and Perfect Groups

	**percent P to F-P**	**percent HCl-5% to H**_**2**_**SO**_**4**_**-5%**	**percent HCl-10% to H**_**2**_**SO**_**4**_**-10%**	**percent HCl-15% to H**_**2**_**SO**_**4**_**-15%**	**percent HCl-20% to H**_**2**_**SO**_**4**_**-20%**	**percent F-HCl-5% to F- H**_**2**_**SO**_**4**_**-5%**	**percent F-HCl-10% to F- H**_**2**_**SO**_**4**_**-10%**	**percent F-HCl-15% to F- H**_**2**_**SO**_**4**_**-15%**	**percent F-HCl-20% to F- H**_**2**_**SO**_**4**_**-20%**
max load (Fu)	31.71	3.67	5.84	6.51	4.91	–4.60	0.78	5.58	0.32
Kp/Ke	8.34	3.05	–7.40	33.64	23.73	16.61	20.75	8.18	9.59
deformation (Δ*f*)	32.91	39.03	3.57	29.23	–7.43	–8.6	–4.02	20.07	25.75
energy dissipation	54.18	41.27	11.30	33.83	–2.17	–13.59	–3.20	24.53	25.98

In H_2_SO_4_ corrosion, the Kp/Ke ratio value decreases considerably compared to HCl corrosion. So, if the joint is exposed to H_2_SO_4_ corrosion, then its semirigidity behavior approaches hinged behavior in the fire condition specimens. Also, the Kp/Ke ratio value decreased by about 31%, according to the F-P specimen of the P specimen. In addition, the deformation capacity value and energy dissipation value decreased by about 39 and 54%, according to the F-P specimen of the P specimen, respectively.

Generally, compared to H_2_SO_4_ corrosion, HCl corrosion has little effect on the load–deformation characteristics of the connections. Also, if corrosion specimens are exposed to fire, then the connections will change from semirigid to hinged.

## 4 Numerical Investigation

Eighteen three-dimensional (3D) numerical models were created using the commercial FE package ABAQUS/standard^41,42^ to evaluate how bolted T-stub connections will behave in various corrosion with fire conditions. The observed cross-sectional dimensions, initial geometric flaws, material characteristics from the coupon tensile tests, and more were all incorporated into the FE model (Table 2).

### 4.1 Element Type and Mesh Size

The entire model was built using the solid element C3D8R (eight-node reduced integration brick element), which can simulate nonlinearities in geometrical and material behavior.

**Figure 12:**
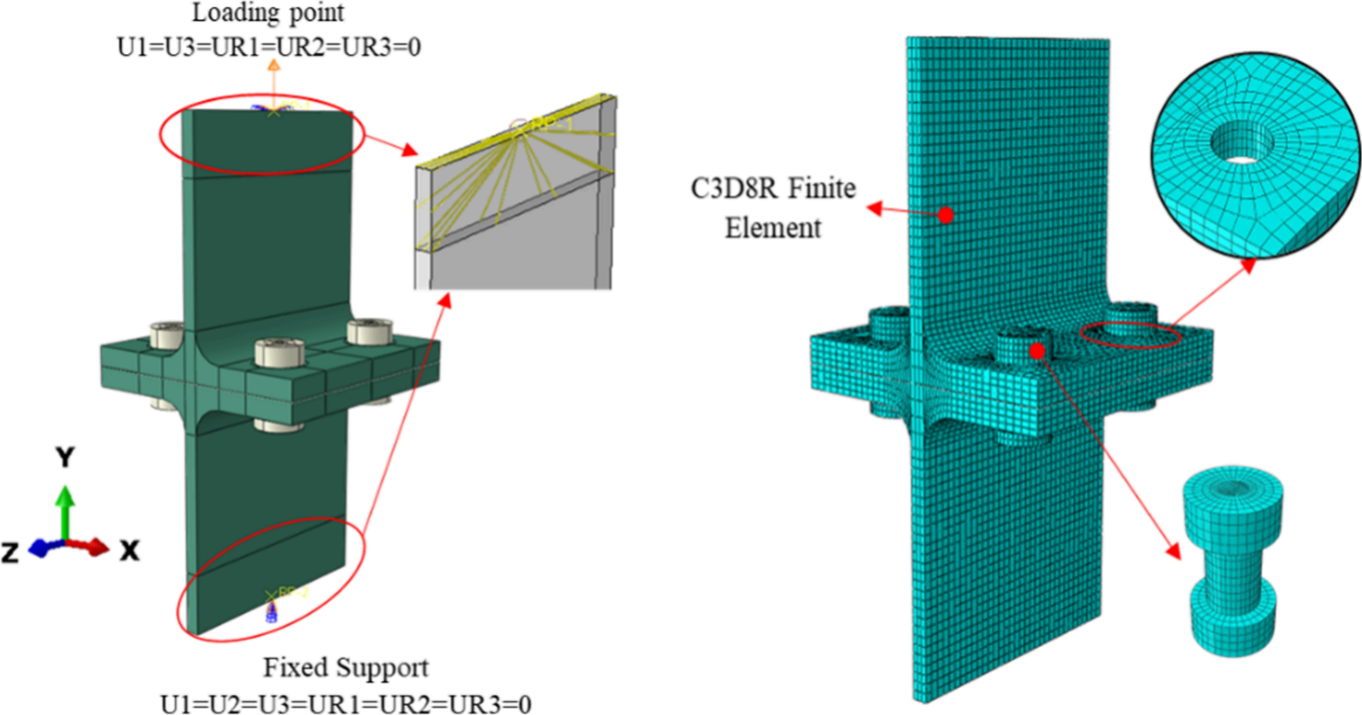
Finite element model of experimental specimens and boundary conditions.

It was determined through mesh sensitivity analysis that a mesh size of 3 mm × 3 mm (length × width) was appropriate for the T-Section connection. Therefore, a mesh size of 2 mm × 2 mm (length by width) was employed for the bolts. For accurate FE analysis, mesh refinement was done around the flange holes, and smaller mesh sizes were used close to the rounded corners (Figure 12).

### 4.2 Boundary Conditions and Loading Procedure

The FE model needed to define the contact pairings, including the interactions between the bolt head and the top surface of the flange and the bolt shank and the inner surface of the bolt hole. A “hard contact” command was used for all of the above contact pairs to allow separation between contacting surfaces. The tangent and standard directions, two orthogonal directions, were used to establish the attributes of each contact pair. A “hard contact” attribute was used to characterize the constitutive relationship in the normal direction, allowing separation after contact. A penalty-based friction model with a 0.3 friction coefficient described the tangential behavior.

The whole FE analysis included four steps. First, a pretension (e.g., 50 kN) was applied to each bolt to restrain it temporarily. Then, for the second and third steps, the model was heated and cooled according to the temperature history measured from the experiment as thermal loading. Finally, in the last step, a monotonically increasing displacement load was applied to the end of the web until it reached the target displacement value, as listed in Table 4.

### 4.3 FE Validation

**Figure 13:**
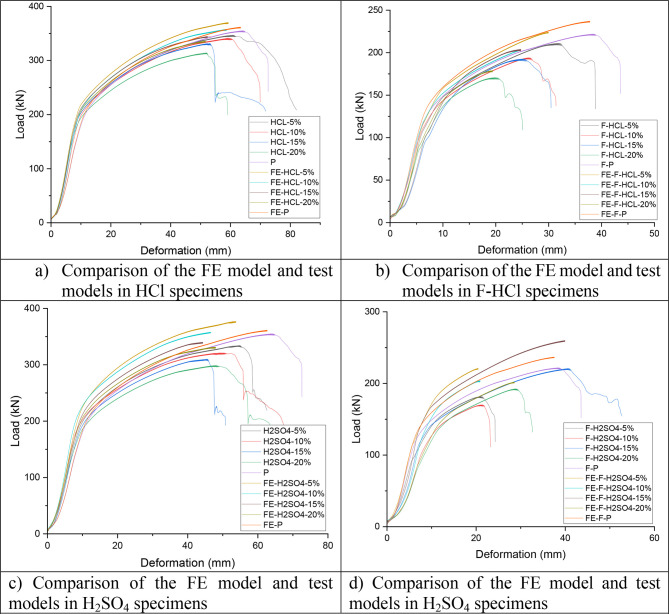
Comparison of the FE model and test models.

**Figure 14:**
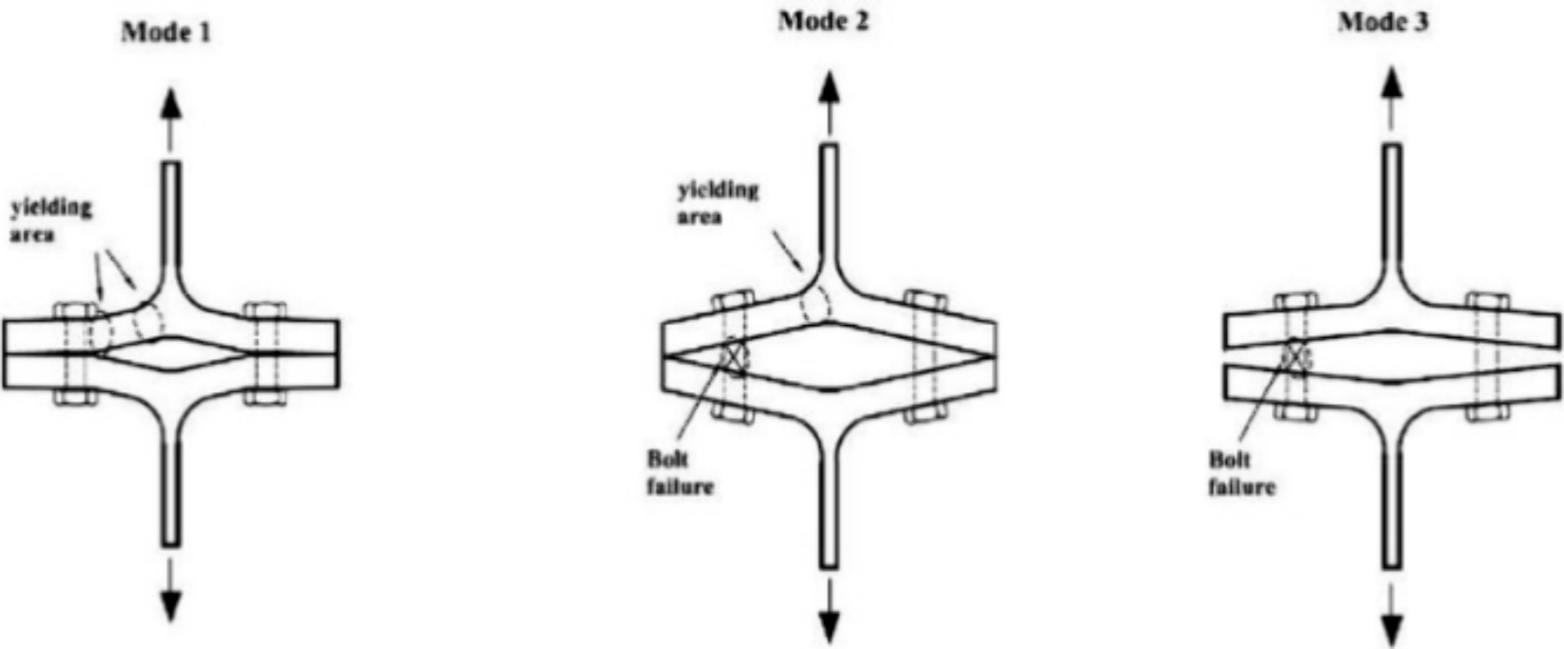
Three failure modes in Eurocode 3.

The FE model was validated against the test results. In Table 5 and Figure 13, a comparison of the test results (*F*_U-EXP_) with the numerical results (*F*_U,FEA_) is shown for all of the investigated specimens. The mean value of the *F*_U,EXP_/*F*_U,FEA_ ratio is 1.00, with the corresponding coefficient of variation (COV) of 0.01. Figure 14 shows the failure mode obtained from the FEA and tests for all test specimens. Overall, the FE results showed reasonable correlations with the experimental results in terms of the moment capacity and failure mode. The differences are attributed to the factors above: imperfections, material properties, actual dimensions of test specimens, and slip of bolts.

**Table 5:** Comparisons of the Moment Capacity of Different Methods

**specimen**	***F***_**U,EXP**_	***F***_**U,FEA**_	***F***_**U,EXP**_**/*F***_**U,FEA**_
HCl-5%	347.10	373.22	0.93
HCl-10%	341.25	359.21	0.95
HCl-15%	331.53	345.34	0.96
HCl-20%	314.28	337.93	0.93
F-HCl-5%	211.21	227.11	0.93
F-HCl-10%	194.11	206.5	0.94
F-HCl-15%	192.67	204.96	0.94
F-HCl-20%	170.80	179.78	0.95
H_2_SO_4_-5%	334.36	384.32	0.87
H_2_SO_4_-10%	321.32	365.13	0.88
H_2_SO_4_-15%	309.96	344.4	0.90
H_2_SO_4_-20%	298.86	335.79	0.89
F-H_2_SO_4_-5%	220.92	283.23	0.78
F-H_2_SO_4_-10%	192.58	240.72	0.80
F-H_2_SO_4_-15%	181.92	221.85	0.82
F-H_2_SO_4_-20%	170.26	179.22	0.95
P	355.20	362.44	0.98
F-P	242.55	260.80	0.93
mean			0.91
COV			0.12

### 4.4 Failure Modes

There are three failure modes in Eurocode 3^36^ for the T-section connections (Figure 14). Mode 1 is the complete flange yielding without bolt failure. Mode 2 is the flange yielding with bolt failure, and Mode 3 is the bolt failure. Figure 14 shows the failure modes, and Table 6 expresses the failure types of all models. Also, the *F*_*y*_/*F_u_* ratio showing the effect of corrosion and fire change on the strength of the connection area is presented in Table 6 (Figure 8). The force values that initiate the flow in the elements in all three groups were obtained from the experimental study. The ratio of displacement Δ_f_ at failure to displacement Δ_*y*_ at yield, which shows the ductility of the connection, is given in Table 6. All Δ_f_/Δ_*y*_ values were compared with the Δ_f_/Δ_*y*_ values of p models, it was observed that there was a decrease in the ductility. When the Δ_f_/Δ_*y*_ ratio of the test elements was exposed to corrosion and fire.

**Table 6:** Yield and Failure Load and Deformation Characteristics and Failure Modes for All Specimens^a^

**specimen**	***F_y_***	**Δ**_*y*_	**F**_y_**/*F***_**u**_	**Δ**_**f**_**/Δ**_*y*_	**failure modes**
HCl-5%	220.87	12.6	0.63	0.15	1
HCl-10%	214.92	12.5	0.63	0.22	1
HCl-15%	207.9	10.5	0.63	0.18	2
HCl-20%	196.53	10.37	0.63	0.22	2
F-HCl-5%	150.156	10.48	0.71	0.27	2
F-HCl-10%	121.52	7.18	0.63	0.29	2
F-HCl-15%	107.55	7.84	0.56	0.32	2
F-HCl-20%	91.31	5.99	0.53	0.24	2
H_2_SO_4_-5%	214.03	11.68	0.64	0.23	2
H_2_SO_4_-10%	208.17	10.65	0.65	0.20	2
H_2_SO_4_-15%	197.04	10.45	0.64	0.26	2
H_2_SO_4_-20%	179.36	9.45	0.60	0.19	2
F-H_2_SO_4_-5%	102.64	6.49	0.46	0.15	2
F-H_2_SO_4_-10%	97.89	7.45	0.51	0.29	2
F-H_2_SO_4_-15%	107.33	6.88	0.59	0.35	2
F-H_2_SO_4_-20%	97.80	8.13	0.57	0.44	2
P	219.18	13.63	0.62	0.23	1
F-P	122.59	6.65	0.51	0.17	2

a *F*_*y*_**:** yield load; *F_u_*: unheated specimen yield load;Δ_f_: failure displacement; Δ_*y*_: yield displacement.

**Figure 15:**
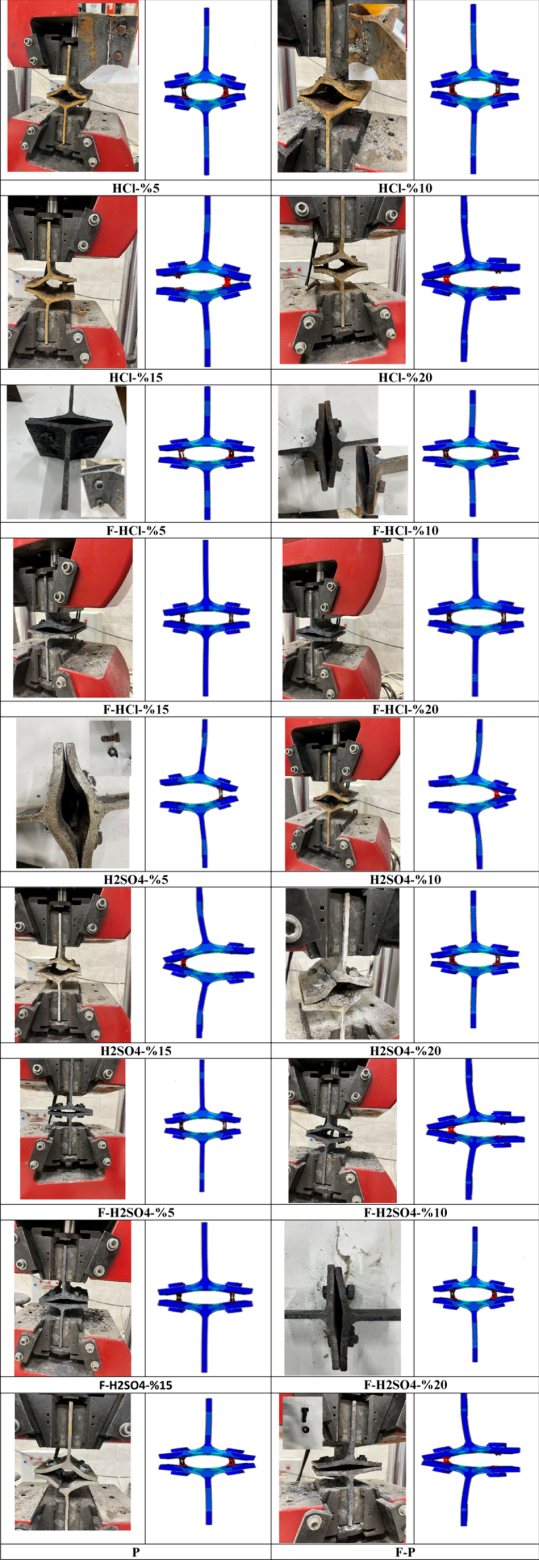
Failure modes of three groups.

Figure 15 and Table 6 show that except for models HCl-5% and HCl-10% vs P, which collapsed in mode 1, all other models collapsed in mode 2. In other words, according to the perfect model, the HCl corrosion value exceeds 10%, the failure mode changes from 1 to 2, and if the T-section connection is exposed to H_2_SO_4_ corrosion, then it occurs as failure mode 2. Also, the failure mode changes from 1 to 2 when compared to the P model for all fired connections. Furthermore, all T-section connections presented a V-shaped form at failure, and the depth of the V-shape of vertically stiffened models is greater than that of the horizontal ones.

Figure 15 shows the FE failure modes of all models. There is also a correlation between the FE failure modes and experimental failure modes of these two completely different ways, which can be taken as further proof of the installation and measurement precisions.

## 5 Conclusions

In this study, the corrosion and fire condition effect that occurs in the T-section connection on the behavior of the connection zone was investigated. The results can be used in industry works in the field of steel construction. The study was carried out with 18 T-section connections with various corrosion (hydrochloric acid and sulfuric acid) in different layers at 5, 10, 15, and 20% corrosion levels, and fire (ISO834) conditions after corrosion have been evaluated and compared. Eighteen connections in the three groups were tested. T-section-bolted joints examined in the study were produced using IPE (300) standard profiles to provide the necessary data for improving Eurocode 3 and the efficient use of residue IPE profiles back to the consumption cycle. The main conclusions of this article can be summarized as follows:

The HCl corrosion value increased from 5 to 20%; the maximum load value decreased by about 10.44%. That is, the maximum load value decreases as the corrosion value increases.
The HCl corrosion with fire condition value increased from 5 to 20%; the maximum load value decreased by 23.65%. The maximum load value decreases as the corrosion with fire value increases.
The F-P specimen is compared with those only exposed to HCl corrosion; the corrosion rate should increase by about 30% to equal the maximum load value of the F-P specimen.
The stiffness ratio (Kp/Ke) values decreased for HCl-5%, HCl-10%, HCl-15%, and HCl-20% specimens, about 15.15, 38.49, 29.30, and 51.34% for the perfect model, respectively. In other words, as the HCl ratio increases, the semirigid behavior will decrease, exhibiting a hinged behavior.
The H_2_SO_4_ corrosion value increased from 5 to 20%; the maximum load value decreased by about 11.88%. That is, the maximum load value decreases as the corrosion value increases.
The H_2_SO_4_ corrosion value increased from 5 to 20%, and the stiffness ratio (Kp/Ke) value decreased by about 66.48%. The stiffness decreases as the H_2_SO_4_ corrosion ratio increases from 5 to 20%. In other words, as the H_2_SO_4_ ratio increases, the semirigid behavior will decrease, exhibiting a hinged behavior.
The maximum load value decreased by about 0.32–6.51%, according to the H_2_SO_4_ specimens of the HCl specimens. In other words, in H_2_SO_4_ corrosion, the maximum load value decreases considerably compared to HCl corrosion. So, if the joint is exposed to H_2_SO_4_ corrosion, then its semirigidity behavior approaches that of hinged behavior.
In H_2_SO_4_ corrosion, the Kp/Ke ratio value decreases considerably compared to HCl corrosion. So, if the joint is exposed to H_2_SO_4_ corrosion, then its semirigidity behavior approaches hinged behavior in the fire condition specimens.
Compared to H_2_SO_4_ corrosion, HCl corrosion has little effect on the load–deformation characteristics of the connections. Also, if corrosion specimens are exposed to fire, then the connections will change from semirigid to hinged.
Except for models HCl-5% and HCl-10% vs P, which collapsed in mode 1, all other models collapsed in mode 2. In other words, according to the perfect model, if the HCl corrosion value exceeds 10%, then the failure mode changes from 1 to 2, and if the T-section connection is exposed to H_2_SO_4_ corrosion, then it occurs as failure mode 2. Also, the failure mode changes from 1 to 2 compared to the P model for all fired connections.
